# Origin of poor doping efficiency in solution processed organic semiconductors†
†Electronic supplementary information (ESI) available: Additional details on sample characterization, quantum chemistry calculations to obtain transition dipole moments of the ions and determine the strength of the Coulomb interaction, two-dimensional correlation analysis has been provided. In addition, this document also contains details of the calculations used to simulate 2D electronic spectra. See DOI: 10.1039/c8sc00758f


**DOI:** 10.1039/c8sc00758f

**Published:** 2018-04-10

**Authors:** Ajay Jha, Hong-Guang Duan, Vandana Tiwari, Michael Thorwart, R. J. Dwayne Miller

**Affiliations:** a Max Planck Institute for the Structure and Dynamics of Matter , Luruper Chaussee 149 , 22761 , Hamburg , Germany . Email: dwayne.miller@mpsd.mpg.de; b I. Institut für Theoretische Physik , Universität Hamburg , Jungiusstraße 9 , 20355 Hamburg , Germany; c The Hamburg Center for Ultrafast Imaging , Luruper Chaussee 149 , 22761 Hamburg , Germany; d Department of Chemistry , University of Hamburg , Martin-Luther-King Platz 6 , 20146 Hamburg , Germany; e The Departments of Chemistry and Physics , University of Toronto , 80 St. George Street , Toronto , Canada M5S 3H6

## Abstract

Doping is an extremely important process where intentional insertion of impurities in semiconductors controls their electronic properties. In organic semiconductors, one of the convenient, but inefficient, ways of doping is the spin casting of a precursor mixture of components in solution, followed by solvent evaporation. Active control over this process holds the key to significant improvements over current poor doping efficiencies. Yet, an optimized control can only come from a detailed understanding of electronic interactions responsible for the low doping efficiencies. Here, we use two-dimensional nonlinear optical spectroscopy to examine these interactions in the course of the doping process by probing the solution mixture of doped organic semiconductors. A dopant accepts an electron from the semiconductor and the two ions form a duplex of interacting charges known as ion-pair complexes. Well-resolved off-diagonal peaks in the two-dimensional spectra clearly demonstrate the electronic connectivity among the ions in solution. This electronic interaction represents a well resolved electrostatically bound state, as opposed to a random distribution of ions. We developed a theoretical model to recover the experimental data, which reveals an unexpectedly strong electronic coupling of ∼250 cm^–1^ with an intermolecular distance of ∼4.5 Å between ions in solution, which is approximately the expected distance in processed films. The fact that this relationship persists from solution to the processed film gives direct evidence that Coulomb interactions are retained from the precursor solution to the processed films. This memory effect renders the charge carriers equally bound also in the film and, hence, results in poor doping efficiencies. This new insight will help pave the way towards rational tailoring of the electronic interactions to improve doping efficiencies in processed organic semiconductor thin films.

## Abstract 2

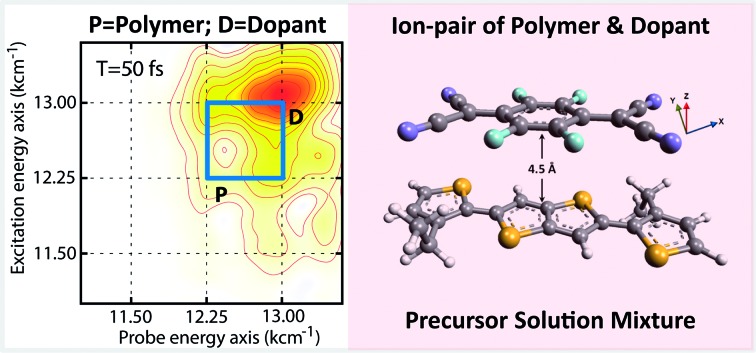
We have employed two-dimensional electronic spectroscopy to reveal detrimental electronic coupling among the ions in precursor solution of molecular-doped polymer, which are retained to the processed films. This memory effect renders the charge carriers to be bound resulting into poor doping efficiency.

Organic semiconductors have attracted enormous attention due to the advantage of convenient large area processing *via* spin coating or printing techniques, and due to their potential in designing custom-tailored molecules to tune optoelectronic properties for organic electronics.^1^ To gain control over the intrinsic charge carrier concentration in organic semiconductors, doping with small molecules and ions is commonly used. Conjugated semiconducting polymers have shown remarkable conductivities as high as ∼105 S cm^–1^ in a solid pellet of polyacetylene^2^ and ∼2000 S cm^–1^ in solution-processed polythiophenes.^3^ Recent experiments have revealed the unforeseen potential of doping and have resolved various issues in organic semiconductors beyond its traditional role of increasing conductivities. This includes organic light emitting diodes,^4^,^5^ solar cells,^6^ thermoelectric generators^7^,^8^ and thin film transistors.^9^ However, despite the initial successes of the doped materials, it remained difficult to precisely ascertain the dominating mechanisms, which should guide the further improvement of the materials' performance.^10^

To obtain higher conductivities, empirical efforts have been developed for selecting appropriate molecular dopants, which are compatible with the network of conducting polymers as organic semiconductors. The choice of the complementary dopant and polymer combination is mainly determined by energy level alignment which is favorable for the charge transfer. Usually, the highest occupied molecular orbital (HOMO) of the donor lies higher in energy than the lowest unoccupied molecular orbital (LUMO) of the acceptor, which works in most cases with few exceptions.^11^ For p-type doping, the successful donor:acceptor (D:A) pair configuration is a polymer:dopant pair. However, the roles are inverted for n-type doping. To achieve doped conducting polymer films over a desired substrate, a convenient approach is based on a processable solution in which a dopant and a polymer material are co-deposited from a solution mixture *via* spin coating.^12^ This technique of D:A mixing in solution leads to the formation of either an ion-pair, where a duplex of interacting charges is formed by electron transfer, or, of a ground state charge transfer complex, where frontier molecular orbitals of the polymer and the dopant hybridize to generate a new set of bonding and antibonding orbitals.^13^ How the small-molecule dopants are incorporated in order to achieve these fundamental interaction mechanisms has been an active area of research.^14^,^15^ Amongst these two interaction mechanisms, ion-pair formation has been identified as the dominant effect, which is operative in doped organic semiconductors. The doping efficiency (*i.e.*, the number of free charge carriers created per dopant molecule) is completely determined by these prevailing interactions. The strength of the Coulomb interaction within the ion-pairs thereby determines the binding energy of the charge carriers generated after the doping process. Thus, it becomes important to decipher the origin and strength of the dominating interactions in the course of the doping process. Interestingly, the optical signatures and dynamics for the ion-pair formation process can even be observed in the solution mixture used for spin-casting.^16^ Although there have been numerous attempts to decipher the nano-morphologies and molecular structures of the doped polymer films using solid-state NMR,^16^ IR/Raman spectroscopy,^17^ small-angle neutron or X-ray scattering,^18^,^19^ electron microscopy^20^ and inelastic neutron spectroscopy,^21^ there is minimal understanding of the strength of the dominant intermolecular interactions prevailing in the precursor solutions, which dictate the incumbent film properties. A direct determination of the intermolecular interaction strength of the ion pair in solution is needed to formulate the guidelines to gain systematic control over the molecular design for doped organic semiconductor to improve the doping efficiency.

In order to develop a specific molecular basis for rational tailoring of molecular interactions in ion pairs within doped polymer materials, we have explored the electronic structure of p-doped thiophene-based conjugated polymer, PBTTT (poly(2,5-bis(3-dodecylthiophen-2-yl)thieno[3,2-*b*]thiophene)) in chlorobenzene as a model system. The dopant used in our studies is tetrafluoro-tetracyano-quinodimethane (F_4_TCNQ). We employed ultrafast nonlinear two-dimensional electronic spectroscopy to probe the electronic interactions amongst the ion pair in the precursor solution mixture. The use of 2D methods provides a means to directly determine the inhomogeneous distribution of ion pair interactions and equally important the degree of interaction as measured by the effect of electronic coupling on the homogeneous lineshape. Our measurements prove the presence of strong electronic coupling within the ion pair already in solution and this distribution corresponds to a spectrally well resolved state. We do not observe a broad distribution of interactions as might be expected for well solvated species in which solvation energies overcome Coulomb interactions. In addition, the electronic coupling between the dopant and polymer strongly perturbs electronic structure of the dopant anion, which is inferred from its modulated decay dynamics. We have developed a theoretical model, which allows us to extract the value of the electronic coupling strength to be ∼250 cm^–1^, which is unexpectedly large. Likewise, it retrieves an intermolecular distance of ∼4.5 Å within the ion-pair distribution. The lineshape indicates a relatively narrow distribution about this charge separated pairs. Using this combination of experimental and theoretical results, a structural model of the ion pair in the precursor solution is established and compared with the postulated model in spin casted films by solid-state NMR^21^ to reveal the retained memory of the precursor solution interactions in the films. We conclude that already at the level of the precursor solution, the electronic interaction in the ion pair is fixed and the film inherits this property, which becomes the decisive parameter for the electronic conductivity in the films. Yet, strategies are available for controlling chemical processing at the level of the precursor solution.

## Results and discussion

### PBTTT/F_4_TCNQ: a model system of ion-pair formation in doped organic semiconductors

**Fig. 1:**
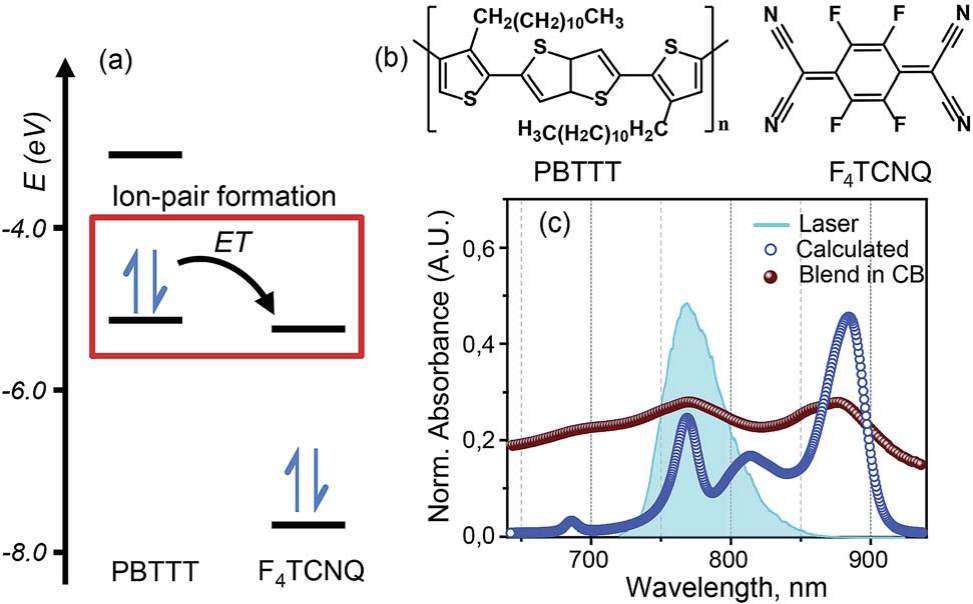
PBTTT/F_4_TCNQ: a model system for the ion-pair in p-doped organic semiconductors. (a) Unperturbed energy diagram depicting the electronic states of PBTTT and F_4_TCNQ, which are involved in the formation of the ion-pair. Electron transfer (ET) from PBTTT to F_4_TCNQ is favored by the sizable energy offset between the ionization energy (IE) of PBTTT (–5.1 eV) and the electron affinity (EA) of F_4_TCNQ (–5.2 eV). The values of the IE and EA of F_4_TCNQ and PBTTT are used from literature report.^16^ (b) Chemical structures of the polymer PBTTT and the dopant, F_4_TCNQ, used in this study. (c) Experimental (brown spheres) and calculated (blue circles) absorption spectra of the PBTTT^+^F_4_TCNQ^–^ ion-pair at room temperature. The vibronic features in the experimental absorption spectrum in chlorobenzene are diagnostic for the F_4_TCNQ anion (D_0_ → D_1_). The blue filled curve represents the laser spectrum used in the two-dimensional electronic spectroscopic measurements which covers the polaron peak, P_2_ of PBTTT at 815 nm (∼12 250 cm^–1^) as well as the second vibronic feature at 769 nm (∼13 000 cm^–1^) corresponding to the F_4_TCNQ anion.

Recent studies of doped PBTTT by F_4_TCNQ have revealed promising values of electrical conductivity from this pair.^16^,^22^ Fig. 1(b) represents the molecular structures of polymer PBTTT and dopant F_4_TCNQ. Polymer PBTTT consists of a thieno [3,2-*b*] thiophene (TT) core having two fused thiophene rings with 6Ï electrons in each monomeric unit. These TT units are in conjugation with thiophene bridges in the backbone which manifests a broad π–π* absorption in the range of 350 to 640 nm with an evident shoulder at 580 nm as shown in the ESI (Fig. S1†).^16^ Dopant F_4_TCNQ on the other hand shows a strong dipolar excitation between 350 to 550 nm in chlorobenzene (blue trace in Fig. S1, ESI†). The solution mixture of dopant and polymer in chlorobenzene (dielectric constant = 5.6) has been prepared using the protocol reported by Chabinyc and co-workers (see Methods section for details).^16^ Black color solution so obtained has 10 wt% of dopant to polymer (molar ratio = 0.25), which corresponds to ∼4 PBTTT repeat units per F_4_TCNQ.^16^ This solution exhibits a distinct structural band in the range of 650 to 1000 nm as shown in Fig. 1 as brown spheres (also in Fig. S1, ESI†). The measured absorption spectrum of the molecular system shows three peaks at 689 nm, 769 and 869 which are characteristic features of F_4_TCNQ anion's HOMO to LUMO transition (D_0_ → D_1_).^24^ These pronounced vibronic structures in F_4_TCNQ anion arises from the Franck–Condon displacements along the C=C stretching vibrational mode of ∼1500 cm^–1^.^31^ The broadening of these vibronic features is caused by the overlapping PBTTT positive polaron features. Molecular dopants are known to interact with the polymer matrix *via* two possible fundamental interaction mechanisms, *i.e.*, ion-pair and ground state charge transfer complex formation.^13^ Since the PBTTT/F_4_TCNQ pair shows diagnostic electronic transitions for the F_4_TCNQ anion in the solution mixture, we can conclude that PBTTT/F_4_TCNQ forms an ion-pair in solution. Our conclusion is also supported by the fact that the electron affinity of the F_4_TCNQ is higher than the ionization energy of the polymer so that one electron can be transferred from the HOMO of the PBTTT polymer to the LUMO of the F_4_TCNQ, as shown in Fig. 1(a).^16^ In the absorption profile of PBTTT/F_4_TCNQ, the spectral feature for PBTTT cation is rendered invisible due to the overlap with the features stemming from the F_4_TCNQ anion.^16^,^22^ The optical transitions associated with the PBTTT cation and the F_4_TCNQ anion are calculated on a constructed model of the ion-pair (details have been mentioned in Theoretical modeling section) by means of density functional theory (DFT) using CAM-B3LYP with the diffuse basis set of cc-pvdz, which revealed the transition dipole moments of the PBTTT and F_4_TCNQ ions' electronic transitions with the magnitude of 3.3 and 6.5 Debye, respectively, in the spectral range of 650 to 950 nm (for details, see the ESI†). The feature at 815 nm (∼12 250 cm^–1^) corresponds to the P2 electronic transition associated with the PBTTT cation (blue circles in Fig. 1). Thus, the optical absorption spectrum of PBTTT/F_4_TCNQ in chlorobenzene is a superposition of contributions from F_4_TCNQ anions and PBTTT positive polarons which signifies the presence of ion-pair formation in the solution phase. These electronic transitions of PBTTT^+^F_4_TCNQ^–^ in chlorobenzene can be capitalized to explore the intermolecular electronic interactions in the ion-pair.

### Two-dimensional electronic spectroscopy (2DES) measurements

**Fig. 2:**
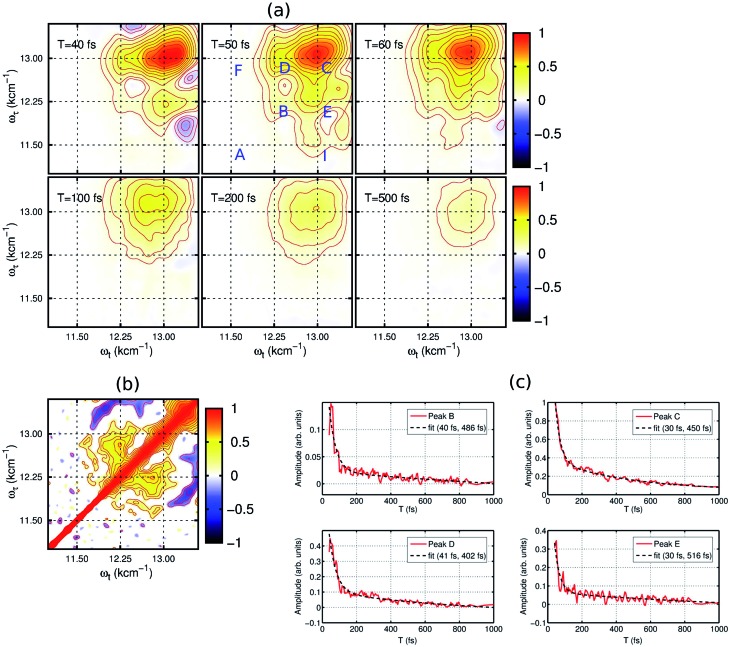
Experimental 2D electronic spectra (real part) of the PBTTT^+^F_4_TCNQ^–^ ion-pair in chlorobenzene at the selected evolution times. (a) Red and blue peaks represent the photo-induced increase and decrease of the transmission due to the ground-state bleach and the excited-state absorption, respectively. The diagonal and off-diagonal peaks are clearly resolved even at room temperature. They are labeled in the spectrum for *T* = 50 fs by capital letters (A, B, C) and (D, E, F, I), respectively. The 2D spectra for early time points show a rich structure and they decay rapidly within the initial *T* = 100 fs, which manifests the decay of the electronic wave packet from the excited state surface back to the ground state *via* the conical intersection. The spectra are normalized to the maximum of the F_4_TCNQ anion bleach signal. (b) Signature of electronic coherence in 2D correlation map. The off-diagonal peaks at (12 250, 13 000) and (13 000, 12 250) cm^–1^ demonstrate the presence of electronic coherence in the excitonically coupled electronic states of the PBTTT^+^F_4_TCNQ^–^ ion-pair in chlorobenzene. (c) Ultrafast decay dynamics and frequency analysis. The red curves show the decay kinetics of the selected peaks (B–E) which correspond to the different location in the 2D electronic spectra shown in (a). All the kinetic traces have been fitted to a bi-exponential function shown as black traces.

2DES is a powerful nonlinear optical spectroscopic technique which provides a correlation between the excitation (*ω*_*τ*_) and the probing wavelength (*ω*_*τ*_) presenting connectivity in different electronic transitions to reveal the electronic coupling between corresponding chromophores.^25^–^27^ This method also provides a direct means to separate inhomogeneous broadening effects from the 2D lineshape to enable a determination of the underlying distribution of nuclear configurations giving rise to the inhomogeneous lineshape. To unveil the interplay of the different electronic transitions and their couplings in PBTTT^+^F_4_TCNQ^–^ ion-pair, we have carried out 2DES measurements at room temperature for different waiting times. The details of the experimental set up and the measurement conditions are described in the Methods section. PBTTT/F_4_TCNQ in chlorobenzene is excited by an ultrashort laser pulse with the pulse width of 16 fs and the associated spectrum is shown as the blue curve in Fig. 1(c). In order to avoid the interference with the 0–0 transition of the F_4_TCNQ anion, the frequency window of the laser spectrum has been judiciously chosen to cover two specific transitions corresponding to the polaron peak of PBTTT at 815 nm (∼12 250 cm^–1^) and the second vibronic feature at 769 nm (∼13 000 cm^–1^) of the F_4_TCNQ anion. Measured 2D spectra (real part) for selected waiting times are shown in Fig. 2(a). Interestingly, the measured 2D spectra for different waiting times show several diagonal and off-diagonal features, which represent the underlying optical transitions and the coupling between them. To identify the individual features, we have marked the diagonal and off-diagonal peaks in the 2DES spectrum for *T* = 50 fs by (A, B, C) and (D, E, F, I), respectively. The diagonal peak A corresponds to the 0–0 transition between the ground and excited state of the F_4_TCNQ anion. Since this transition is only weakly absorbed within the laser spectrum as shown in Fig. 1 (blue filled curve), it does not show an appreciable magnitude in the 2DES spectra. The diagonal peak B at *ω*_*τ*_ = 12 250 cm^–1^ is a signature of the PBTTT positive polaron.^22^,^28^ Peak C at *ω*_*τ*_ = 13 000 cm^–1^ is a strong positive bleach feature and corresponds to the transition to the higher vibrational level of the excited state, D_1_ of the F_4_TCNQ anion. It is worth mentioning here that unlike the linear absorption spectrum of PBTTT^+^F_4_TCNQ^–^ in chlorobenzene in Fig. 1, 2DES is able to resolve the features corresponding to the PBTTT positive polaron as peak B despite its weak transition dipole strength. In addition to the diagonal peaks, 2DES spectra also display clear off-diagonal features. The off-diagonal peaks D and E demonstrate the existence of a noticeable electronic coupling between the transitions corresponding to B and C. Thus, these off-diagonal features are direct evidence of the electronic interaction between the F_4_TCNQ anion and the PBTTT cation. The other set of off-diagonal peaks F and I manifests vibronic coupling between the F_4_TCNQ anion's transitions corresponding to A and C. The absence of any signal at the position F is probably caused by the additional overlapping excited-state absorption features. It is important to realize that the diagonal and off-diagonal peaks are clearly resolved in the 2D spectra even at room temperature. The specific shape of the 2D spectrum with well defined off diagonal couplings shows a small inhomogeneity of the F_4_TCNQ within the environment of the polymer as a host. The small inhomogeneity of the underlying transition is a direct reflection of the exceptionally homogeneous intermolecular interactions of the F_4_TCNQ anion through the polymer backbone. Thus, the 2DES spectra clearly resolve the electronic coupling between the polymer cation and the dopant anion within the ion-pair, which are homogeneously interacting in the solution.

The dynamics of the different states can be observed by the time evolution of the 2D electronic spectra, as shown in Fig. 2(a). At *T* = 40 fs, the diagonal peaks (B and C) and the off-diagonal peaks (D and E) are clearly evident with strong intensities. Additionally, negative features corresponding to excited-state absorption can also be seen in different frequency regions, which overlap with the strong off-diagonal features. With the time evolution, we observe an increase in the magnitude of the off-diagonal peak I as shown in the 2DES spectrum for *T* = 50 fs in Fig. 2(a). It clearly signifies the vibronic coupling between the A and C transitions of the F_4_TCNQ anion. From the waiting time *T* = 100 fs, we can observe a rapid decrease in the magnitude of the different spectral features constituting the 2D maps. In fact, the 2D map at *T* = 500 fs shows only one diagonal peak C corresponding to the F_4_TCNQ anion with an extremely small magnitude. Verlet and co-workers have shown that the lower energy excited state in this F_4_TCNQ anion relaxes within 200 fs *via* a conical intersection.^24^ Based on this, we can conclude here that in the PBTTT^+^F_4_TCNQ^–^ ion-pair, the electronic wave packet on the excited state surface of the F_4_TCNQ anion undergoes a fast decay back to the ground state *via* the conical intersection.

To gain further insight into the electronic interaction amongst the ions, we performed a 2D correlation analysis of the residuals obtained by subtracting the underlying global kinetics from the real part of the measured 2D spectra (see the ESI† for the details of the analysis). The 2D correlation map so obtained is shown in Fig. 2(b). It clearly shows two symmetric off-diagonal peaks at (12 250, 13 000) and (13 000, 12 250) cm^–1^, which perfectly complements the electronic energy gap of the transitions in the PBTTT^+^F_4_TCNQ^–^ ion-pair. Earlier reports from different groups have demonstrated that the existence of positive correlations in this 2D correlation analysis is a unique signature of electronic coherence.^29^,^30^ Based on this phase correlation, we confirm the presence of electronic coherence in coupled PBTTT^+^F_4_TCNQ^–^ in solution. However, we do not observe any evidence of long-lived electronic coherence or that coherence would be prolonged by the vibronic coupling. This is possibly due to the presence of fast decay channels for the excited state wave packet to relax back to the ground state. Secondly, we can quantify the static disorder of 350 cm^–1^, which the polaron experiences (see ESI† for details). This inhomogeneous broadening gives a relative measure of the coupling of the electronic transitions to the bath. Based on this degree of coupling and resulting site distribution, we can attribute the fast decoherence due to this relative strong interaction with the bath. In addition, the off-diagonal blue peaks manifest the vibronic coupling of 0–1 transitions to the higher ones for the F_4_TCNQ anion.

In addition, the kinetics of the decay of the photoexcited ions can also be modulated by the presence of interacting counter-ions, such that the dynamics will reflect the perturbed electronic structure of the constituent ions. To quantify the deactivation dynamics of the dopant F_4_TCNQ anion within the ion-pair in chlorobenzene, we have carried out a detailed analysis of the kinetics of selected diagonal and off-diagonal peaks. The results are shown in Fig. 2(c). Each kinetic trace has been fitted with two exponential decay functions and the retrieved time-constants are mentioned in the respective kinetic curves. All the traces clearly show one fast decay component with a time constant of <40 fs along with the other longer decay time component of ∼500 fs. It is important to point out that Verlet and co-workers have reported a decay time constant of ∼200 fs for the F_4_TCNQ anion in the gas phase.^24^ Although there has been no report on excited state lifetimes of the D_1_ state in solution for the F_4_TCNQ anion, transient absorption measurements of the TCNQ anion have revealed an increased decay time constant of ∼2 ps (alongwith a minor component of ∼20 ps) for the D_1_ state in the solution phase as compared to 650 fs in the gas phase^23^ In our study, we observe a bi-exponential decay with an ultrafast decay with a time constant of ∼30 fs for the D_1_ state of the F_4_TCNQ anion within the ion-pair. This ultrafast decay component hints towards new possible relaxation pathway(s) for the excited state of the F_4_TCNQ anion in the solution mixture. In addition, peak B which corresponds to the polymer polaron, also shows an ultrafast decay time constant of 40 fs along with a slower component of ∼0.5 ps. Thus, the presence of a strong electronic interaction between ion-pairs greatly modulates the decay dynamics of the constituting ions.

### Theoretical modeling

**Fig. 3:**
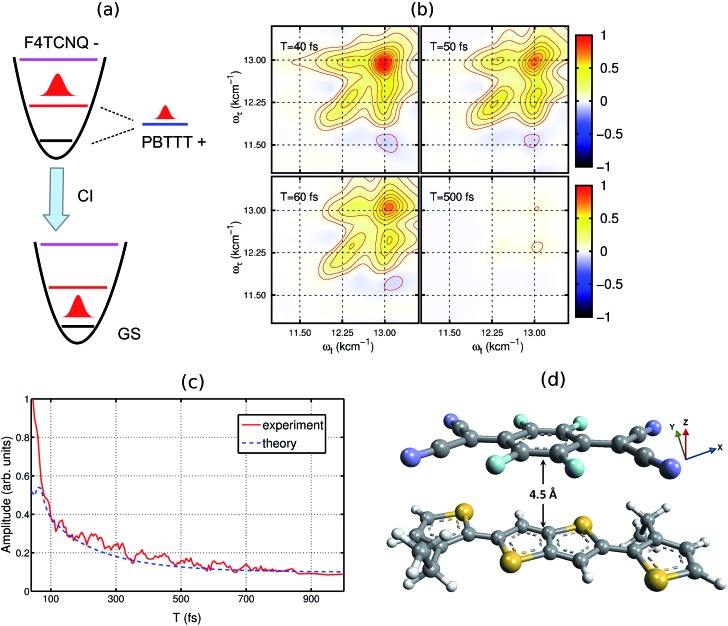
Theoretical model used for calculating the 2DES spectra. (a) The energy diagram of the proposed model. The vibrational dynamics of F_4_TCNQ is described by a harmonic oscillator. The polaron formation is modeled by one electronic state which is strongly coupled to the lattice vibrations. (b) Simulated 2D electronic spectra for different waiting times. (c) Comparison of the experimental and the simulated decay dynamics. (d) Proposed molecular configuration of the ion-pair with an intermolecular distance of ∼4.5 Å. For the modeling, cation and anion of ion-pair interact vai π-stacking interaction as proposed by Chabinyc and co-workers.^16^ To treat the dipolar environment properly, the solvent has been modeled as a thermal bath of harmonic oscillators and the dipole–dipole interaction between the ion-pair and solvent has been included in the system-bath interaction.

To understand the experimental data on the quantitative level, we have modeled the optical absorption and 2D electronic spectra of the PBTTT^+^F_4_TCNQ^–^ ion-pair. In order to calculate these spectra, we have constructed a model which is shown as the unperturbed energy diagram in Fig. 3(a). For simplification, we assume that the vibrational mode of 1500 cm^–1^ corresponding to C=C stretching in the F_4_TCNQ anion is harmonic. As discussed earlier, Franck–Condon displacements along this mode cause pronounced vibronic signatures in the absorption spectrum. The vibronic coupling strength (given by the Huang–Rhys factor, *S* = 0.5) was estimated from the absorption spectrum of the TCNQ anion reported earlier.^31^ To include the contribution of the observed positive polaron peak B marked in Fig. 2(a), we have added one electronic state with the site energy of 12 250 cm^–1^ in our model, which is assumed to describe the P_2_ state of the PBTTT cation. In order to attain the small magnitude of peak B as observed in the absorption and the 2D spectra, a large static disorder (*Δ* = 350 cm^–1^) is included for the polymer chain of PBTTT. The bold arrow labeled as ‘CI’ in Fig. 3(a) indicates the deactivation pathway of the electronic wave packet from the excited state back to the ground state *via* the conical intersection. We model this deactivation dynamics in terms of a Lindblad master equation with appropriate decay rates. To calculate the absorption and 2D electronic spectra, we assume that cation and anion of the ion-pair interact due to the parallel π-stacking, which was earlier proposed by Chabinyc and co-workers for PBTTT/F_4_TCNQ in films.^16^ It is important to mention here that Sirringhaus and co-workers have proposed a different orientation of the dopant in doped-polymer matrix obtained by solid-state diffusion method.^22^ For solution processed films, π-stacking is the dominant interaction mechanism as shown by Chabinyc and co-workers using a combination of solid-state NMR and synchrotron X-ray scattering.^16^ To account for the dipolar environment, the system-bath model has been developed which include the dipole–dipole interactions between ion-pair and solvent. Further details on the model can be found in the ESI.†

Based on the model, we have calculated the absorption spectrum shown in Fig. 1(c) (blue circles). We observe that the positions for all peaks agree with the experimental absorption spectrum. Moreover, we further examine the validity of the model by calculating 2D electronic spectra and comparing them to the experimental data. The calculated 2D electronic spectra for different waiting times are shown in Fig. 3(b). The obtained 2D maps clearly reproduce the experimentally observed diagonal and off-diagonal features along with the population kinetics for the excited states. For comparison, we have examined the decay traces at different points of the calculated 2D spectra and compared them with experimental decays, as shown in Fig. 3(c). The calculated kinetic traces show a fast decay component with a time constant of ∼50 fs, together with an additional slow decay component as also observed in the experimental kinetic traces. This further confirms the validity of our model of the ion-pair. Based on these simulations, we successfully retrieve a strong electronic coupling of ∼250 cm^–1^ amongst the ions of the ion-pair. It is worth mentioning that in spite of a strong electronic coupling, we do not observe any evidence of long-lived electronic coherence in our simulations (details are discussed in Section V of the ESI and in Fig. S3†).^32^ Using the results of our calculations, we can easily retrieve the distance between the cation and the anion of the ion-pair in the precursor solution obtained by quantum chemistry calculations. The intermolecular distance in the ion-pair is calculated to be ∼4.5 Å, as shown in Fig. 3(d) (details of the structure and calculations are provided in the ESI†).

### Implications for the molecular doping process

Molecular doping in organic semiconductors is achieved by three principle methods: (1) solution processing, where the dopant is co-deposited with the polymer from the solution; (2) solution sequential processing, where the dopant is spin-casted on top of a pre-deposited polymer film; and, (3) solid-state diffusion, where the dopant is evaporated on top of a pre-deposited polymer film by spin coating. Ion-pair formation is attained at different stages in these methods. In the solution processing method, the ion-pair can be observed in the precursor solution itself whereas in the other methods, it is attained after diffusion of the dopant inside the pre-ordered polymer lamellar microstructure. Our study reveals that the distance between the constituent ions in the precursor solution is ∼4.5 Å which is close to the values of ∼4.0 Å observed in solid-state NMR measurements for solution processed films.^21^ We can infer that the dopant interaction with the polymer is essentially not modified during the evolution of the doped-polymer film microstructure from the precursor solution. Thus, our measurements provide direct evidence of a strong electronic coupling amongst the ions of the ion-pair in the p-doped organic semiconductor in solution. This strong electronic coupling is a manifestation of coulombic interaction that renders the charge carriers on the polymer matrix to be bound. Thus, the strong electronic coupling between dopant and polymer contributes to the origin of poor doping efficiency, which, in turn, necessitates the use of higher dopant concentration to achieve a substantial free carrier concentration. But at higher doping ratios, the Fermi level is shown to be pinned by tailing states in the transport gap which leads to the saturation effects.^33^ Our results emphasize that in order to obtain a high doping efficiency by a desired dopant:polymer interaction for solution processed films, control lies at the stage of the precursor solution. Thus, it will be important to formulate a quantitative model of the structure–function relationship, which takes the inter-ionic interactions into account in order to predict charge mobilities in doped organic semiconductors. To better formulate this structure–function relationship, we will extend our studies to additional polymer-dopant systems in future. Definite tests of this concept will involve specific comparisons of doping efficiencies correlated to experimentally determined electronic couplings as a quantitative measure of the inter-ionic interactions.

## Conclusions

Using two-dimensional electronic spectroscopic measurements, we have provided direct experimental evidence of strong electronic coupling within the ion-pairs of p-doped organic semiconductors in solution, which is manifest by the well-resolved off-diagonal peaks in the 2D spectra. Our theoretical model simulates the experimental data and unveils an electronic coupling strength of ∼250 cm^–1^ within the ion-pair. The presence of this unexpectedly strong electronic coupling is also perceived by the modulation of the decay times constant of the D_1_ state of the dopant F_4_TCNQ anion, which is a manifestation of Coulomb interaction with the counter ion. Regarding the structure, we have retrieved an intermolecular distance of ∼4.5 Å between the ions of the ion-pair in solution, which is similar to the reported value for the solution processed films. Thus, the dopant–polymer interaction, which crucially determines the doping efficiencies in doped organic semiconductors, remains essentially unchanged during the microstructural evolution of the doped polymer film from the precursor solution. Our work thereby provides a new level of understanding of the ion-pair in doped polymer systems, which underscores the importance of controlling the intermolecular interactions already in the solution phase to obtain the desired properties in solution-processed films. We see synthetic means to sterically limit close electrostatic interactions as one strategy to weaken this interaction and better enable thermal population of high mobility carrier states to satisfy doping requirements.

## Materials and methods

### Sample preparation

Chlorobenzene solvent and F_4_TCNQ were purchased from Sigma-Aldrich. Polymer PBTTT with *M*_w_ of 40 000–80 000 was purchased from Lumtec, Taiwan. All the materials and solvent were used as received. Blend of PBTTT and F_4_TCNQ was prepared using earlier reported protocol^16^ with slight modification. Firstly, 10 mg ml^–1^ of orange-red colored solution of F_4_TCNQ was prepared in chlorobenzene. Since the high concentration solution of F_4_TCNQ precipitates at room temperature, we heated the solution to 150 °C for 30 min and then kept at 110 °C. The red color solution of PBTTT in chlorobenzene was prepared using the same temperature conditions by adding 10 mg of PBTTT in 1 ml of chlorobenzene. On addition of the F_4_TCNQ solution and PBTTT solution at 110 °C, the color of the solution changes to wine red indicating the formation of ion-pairs in chlorobenzene which was further confirmed by absorption measurements.

### 2D electronic measurements and experimental conditions

Details of the experimental setup have already been described in earlier reports from our group.^32^ Briefly, an all-reflective 2D spectrometer based on a diffractive optic (Holoeye) with a phase stability of *λ*/160 has been used for these measurements.^34^ ∼16 fs pulses for the 2D electronic measurements have been generated by compressing the laser beam from a home-built nonlinear optical parametric amplifier (NOPA) to the Fourier transform limit using the combination of a prism pair and a deformable mirror (OKO Technologies). The NOPA is pumped by a commercial femtosecond laser Pharos from Light Conversion. The temporal profile of the compressed beam was characterized by frequency-resolved optical grating (FROG) measurements and the obtained FROG traces were analyzed using a commercial program FROG3 (Femtosecond Technologies). A broadband spectrum so obtained carried a linewidth of ∼100 nm (FWHM) and was centered at 13 000 cm^–1^ which covered the polaron peak, P_2_ of PBTTT at 815 nm (∼12 250 cm^–1^) as well as the second vibronic feature at 769 nm (∼13 000 cm^–1^) corresponding to the F_4_TCNQ anion. An additional component is the Sciencetech spectrometer model 9055 which is coupled to CCD linear array camera (Entwicklungsburo Stresing). The 2D spectra for each waiting time *T* were collected by scanning the delay time *τ* = *t*_1_ – *t*_2_ in the range of [–128 fs, 128 fs] with a delay step of 1 fs. At each delay step, 200 spectra were averaged to reduce the noise ratio. The waiting time *T* = *t*_3_ – *t*_2_ was linearly scanned in the range of 1 ps with steps of 5 fs. The energy of the excitation pulse is attenuated to 10 nJ with 1 kHz repetition rates for all the measurements. Three pulses are focused on the sample with the spot size of ∼80 μm and the photon echo signal is generated at the phase-matching direction. A fresh sample solution was prepared for each time-resolved measurement. The sample was filtered using a 0.2 μm filter to minimize light scattering, and then sealed in a 1 mm quartz cell (Starna). To avoid the possibility of sample degradation, the cell in the 2D setup was placed on a precise 2D translator and moved at a speed of ∼20 cm s^–1^ in both directions. Absorption spectrum of the sample was measured before and after the measurements using a Shimadzu spectrometer (UV-2600), and no noticeable change was observed.

### Data analysis

2D spectra have been retrieved from the measured photon-echo (PE) signals using the earlier reported procedure.^26^ Briefly, the PE-traces were extracted from the heterodyne signals and transformed along the delay axis *τ* using numerical Fourier transforms which provides frequency axis, *ω*_*τ*_. The observation axis of the spectra corresponding to spectrometer's wavelength scale, were interpolated to the equally-spaced frequencies *ω*_*τ*_. Using the “invariant theorem”,^26^ phasing of the obtained 2D spectra was performed which provide the correct delay of the local oscillator (LO)-filter used. Phasing of the PE heterodyne signal was performed based on the PP signal at the particular waiting time. The stability of the 2D measurements has been examined by the LO delay.

### Theoretical calculations

The charge-neutral molecular systems, PBTTT and F_4_TCNQ are initially optimized by semi-empirical methods and the obtained structures are further optimized by density functional theory. The subsequent molecular structures of the PBTTT cation and the F_4_TCNQ anion are obtained by using CAM-B3LYP with the diffuse basis of cc-pvdz. The associated optical transitions are calculated and the transition dipole moments of the PBTTT cation and the F_4_TCNQ anion are obtained with the magnitude of 3.3 and 6.5 Debye, respectively. The optimized molecular structures are shown in the ESI as Fig. S2.† To further quantify the experimental results, we assume that cation and anion of the ion-pair interact in the parallel π-stacking orientation, which was earlier proposed by Chabinyc and co-workers for PBTTT/F_4_TCNQ in films.^16^ We have calculated the absorption and the 2D electronic spectra using a model shown in Fig. 3(a). The first-order correlation function of the transition dipole is calculated by the time non-local (TNL) master equation.^35^,^36^ In our system-bath model, dipolar solvent has been modeled as a thermal bath of harmonic oscillators and the dipole–dipole interaction between ion-pair and solvent has been taken into account as the system-bath interaction. In order to calculate the 2D spectra, time-dependent master equations are calculated using TNL method and the photon-echo signal is obtained by selecting the phase-matching direction.^37^ Complete details of the calculations are given in the ESI.†

## Conflicts of interest

The authors declare that they have no competing financial interests.

## Supplementary Material

Supplementary information
