# Supplementary material for: Origin of poor doping efficiency in solution processed organic semiconductors
Source: Chem Sci. 2018 Apr 10;9(19):4468–76. doi: 10.1039/c8sc00758f (PMC5956981; doi:10.1039/c8sc00758f)
Supplement: Supplementary file 1 [file SC-009-C8SC00758F-s001.pdf]

Electronic Supplementary Material (ESI) for Chemical Science.
This journal is © The Royal Society of Chemistry 2018

Supplementary Information

Origin of Poor Doping Efficiency in Solution Processed Organic Semiconductors

Ajay Jha[1], Hong-Guang Duan[1,2,3], Vandana Tiwari[1,4], Michael Thorwart[2,3], and R. J. Dwayne Miller[1,3,5]
[1] *Max Planck Institute for the Structure and Dynamics of Matter, Luruper Chaussee 149, 22761 Hamburg, Germany*
[2] *I. Institut für Theoretische Physik, Universität Hamburg, Jungiusstraße 9, 20355 Hamburg, Germany*
[3] *The Hamburg Center for Ultrafast Imaging, Luruper Chaussee 149, 22761 Hamburg, Germany*
[4] *Department of Chemistry, University of Hamburg, Martin-Luther-King Platz 6, 20146 Hamburg, Germany*
[5] *The Departments of Chemistry and Physics, University of Toronto, 80 St. George Street, Toronto Canada M5S 3H6*
(Dated: January 23, 2018)

In this Supplementary Information, we provide additional details on the absorption spectra of neutral PBTTT and the F_4TCNQ molecules and on the quantum chemistry calculations used to obtain the transition dipole moments of PBTTT$^+$ and the anion F_4TCNQ$^-$. We also provide the details of the calculations to determine the strength of the Coulomb interaction in the ion-pair. In addition, we describe the two-dimensional (2D) correlation analysis of the measured 2D electronic spectra. Finally, we elaborate on our theoretical modeling and on the calculations used to simulate the 2D electronic spectra which have been compared to the experimental data.

I. STEADY STATE ABSORPTION SPECTRA OF PBTTT, F_4TCNQ AND THE ION-PAIR

We have measured the absorption spectra of PBTTT, of F_4TCNQ, and of the ion-pair in chlorobenzene. The measured spectra are shown in Fig. S1.

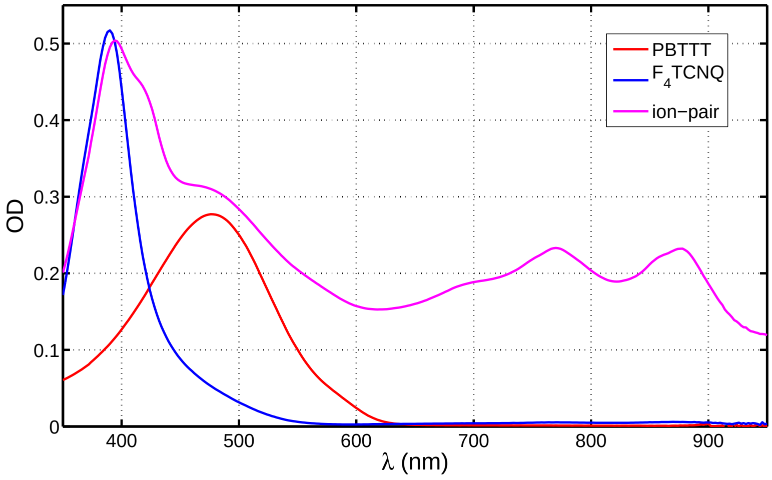

FIG. S1. Absorption spectra of neutral PBTTT (red), F_4TCNQ (blue) and their ion-pair (purple) in chlorobenzene.

II. MOLECULAR STRUCTURE CALCULATION OF THE ION-PAIR

In this section, we describe the quantum chemistry calculation used for the structural optimization and the calculation of the Coulomb interaction of the PBTTT cation and the F_4TCNQ anion using the Gaussian09 package [1]. At the beginning, the molecular structures of the PBTTT cation and the F_4TCNQ anion are initially optimized by semi-empirical methods. The obtained structures are further optimized by DFT calculations (CAM-B3LYP/cc-pvdz). Subsequently, the transition dipole moments between the ground and the excited states are calculated by the TDDFT

approach which provided the magnitude of 3.3 Debye for the PBTTT cation and 6.5 Debye for the F_4TCNQ anion. The corresponding directions of the transition dipole moments are shown in Fig. S2.

On the basis of the theoretical modeling which is described below in Section IV, we obtain the strength of the Coulomb interaction of $\sim 250\ \mathrm{cm}^{-1}$. Thus, we can determine the distance between the cation and the anion based on the calculation of the distribution of the atomic partial charges. The calculational details can be found in Ref. [2]. The initial parallel π-stacked configuration for the ion-pair has been chosen in light of the proposed orientation in films using solid-state NMR measurements [3]. With varying distance between the ions, we find that the distance of 4.5 Å fits best to rationalize the Coulomb interaction of $250\ \mathrm{cm}^{-1}$ between the cation and the anion.

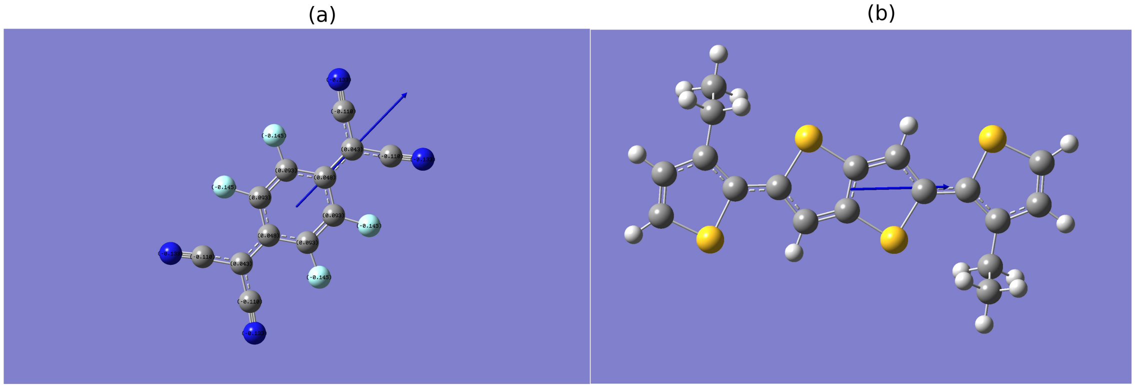

FIG. S2. Molecular structures of the PBTTT cation (a) and the F_4TCNQ anion (b). The directions of their respective transition dipole moments are indicated by the blue arrows.

III. 2D CORRELATION ANALYSIS

To verify the origin of the oscillations observed in the 2D electronic spectra, we have performed a cross-correlation analysis of the residuals across $\omega_\tau = \omega_t$. The residual $\mathrm{R}(\omega_\mathrm{t}, \omega_\tau, \mathrm{T})$ is obtained by subtracting the globally fitted kinetics from the real part of the total 2D electronic spectra. Then, we calculate the correlation coefficients C between two residuals of a pair of conjugated spectral positions in the delay time window up to 2 ps. The delay time steps are equally distributed with intervals of 10 fs. The correlation coefficients are defined as

$$\mathrm{C}(\omega_\mathrm{t}, \omega_\tau) = \mathrm{corr}_\mathrm{T}\{\mathrm{R}(\omega_\mathrm{t}, \omega_\tau, \mathrm{T}), \mathrm{R}(\omega_\tau, \omega_\mathrm{t}, \mathrm{T})\}\,, \tag{S1}$$

where the correlation is evaluated with the respect to T.

IV. THEORETICAL MODELING AND SPECTROSCOPIC CALCULATIONS

A. Time-nonlocal quantum master equation

For the numerical simulations of the linear and 2D spectra, we have applied the time non-local quantum master equation [4, 5]. The method is well established and we briefly summarize it here for the benefit of the reader. The time evolution of the total density matrix $\rho(t)$, which includes system and bath, is governed by the Liouville-von Neumann equation with the Liouville superoperator $\mathcal{L}$ according to ($\hbar = 1$)

$$\dot{\rho} = -i[H_\mathrm{tot}, \rho] = \mathcal{L}\rho\,. \tag{S2}$$

The total Hamiltonian $H_\mathrm{tot} = H_\mathrm{s} + H_\mathrm{b} + \lambda H_\mathrm{sb} + \lambda^2 H_\mathrm{ren}$ includes the system, the bath, and the interaction and renormalization terms. For the system with a single degree of freedom x and the bath consisting of an ensemble of harmonic oscillators $H_\mathrm{b} = \sum_{j=1}^{N}\left[p_j^2/(2m_j) + m_j\omega_j^2 x_j^2/2\right]$, the coupling between them is expressed in the form $H_\mathrm{sb} = f(x)\sum_{j=1}^{N} c_j x_j$ with some real function $f(\cdot)$.

The projection scheme of Nakajima and Zwanzig [6] separates the dynamics of the system and the bath. The bath is assumed to be in a thermal state represented by the canonical density operator $\rho_\mathrm{b}^\mathrm{eq} = \exp(-\beta H_\mathrm{b})$ with a given

temperature $T = (k_B\beta)^{-1}$. Applying the projector $P = \rho_{\rm b}^{\rm ER}{\rm tr}_{\rm b}$ with ${\rm tr}_{\rm b}\rho_{\rm b}^{\rm eq} = 1$ and its orthogonal complement $Q = 1 - P$ yields a formally exact quantum master equation for the time evolution of the reduced system density operator $\rho_{\rm s}(t) = {\rm tr}_{\rm b}\rho(t)$ [4] in the form of

$$\begin{aligned} \dot\rho_{\rm s}(t) &= \mathcal{L}_{\rm s}^{\rm eff}\rho_{\rm s}(t) + \int_0^t K(t,t')\rho_{\rm s}(t') + \Gamma(t), \\ \mathcal{L}_{\rm s}^{\rm eff} &= \mathcal{L}_{\rm s} + \lambda\, {\rm tr}_{\rm b}\mathcal{L}_{\rm sb}\rho_{\rm b}^{\rm eq} + \lambda^2\mathcal{L}_{\rm ren}, \\ K(t,t') &= \lambda\, {\rm tr}_{\rm b}\mathcal{L}_{\rm sb}\left(\mathcal{T}e^{\int_{t'}^t Q\mathcal{L}dt''}\right)Q(\mathcal{L}_{\rm b} + \lambda\mathcal{L}_{\rm sb})\rho_{\rm b}^{\rm eq}, \\ \Gamma(t) &= \lambda\, {\rm tr}_{\rm b}\mathcal{L}_{\rm sb}\left(\mathcal{T}e^{\int_0^t Q\mathcal{L}dt''}\right)Q\rho_{\rm tot}(0)\,. \end{aligned} \tag{S3}$$

Here, $\rho_{\rm tot}(0)$ is the total density operator of system and bath at initial time $t = 0$. The Liouville superoperators $\mathcal{L}_{\rm s}$, $\mathcal{L}_{\rm sb}$ and $\mathcal{L}_{\rm ren}$ are associated with corresponding Hamiltonian operators. Moreover, $\mathcal{L}_{\rm s}^{\rm eff}\cdot = -i[H_{\rm s} + H_{\rm ren}, \cdot]$ and $\mathcal{T}$ is the time ordering operator [7]. Next, we expand the correlated thermal equilibrium state up to first order in the overall system-bath coupling strength λ. This yields

$$\rho^{\rm eq} \approx \frac{1}{Z_{\rm s}}\frac{1}{Z_{\rm b}}e^{-\beta(H_{\rm s}+H_{\rm b})} - \lambda\frac{1}{Z_{\rm s}}\frac{1}{Z_{\rm b}}\int_0^\beta d\beta' e^{-(\beta-\beta')(H_{\rm s}+H_{\rm b})H_{\rm sb}^{(1)}e^{-\beta'(H_{\rm s}+H_{\rm b})}}\,, \tag{S4}$$

with the respective partition functions $Z_{\rm tot} = {\rm tr}\exp(-\beta H_{\rm tot})$, $Z_{\rm b} = {\rm tr}_{\rm b}\exp(-\beta H_{\rm b})$, and $Z_{\rm s} = {\rm tr}_{\rm s}\exp(-\beta H_{\rm s})$. Next, we take the trace over the system degrees of freedom on both sides of Eq. (S4) and obtain

$$\rho_{\rm b}^{\rm eq} = \frac{1}{Z_{\rm b}}e^{-\beta H_{\rm b}} + \frac{\lambda\chi}{Z_{\rm b}}\int_0^\beta d\beta' e^{-(\beta-\beta')H_{\rm b}}\left(\sum_{i=1}^N c_i x_i\right)e^{-\beta' H_{\rm b}}\,. \tag{S5}$$

Here, $\chi = (1/Z_{\rm s}){\rm tr}_{\rm s}\left[f(x)e^{-\beta H_{\rm s}}\right]$ with the coupling function $f(x)$ defined below Eq. (S2). The well-known bath correlation function

$$c(t) = \int_{-\infty}^{\infty}\frac{d\omega}{2\pi}J(\omega)\cos(\omega t)\coth\left(\frac{\beta\omega}{2}\right) - i\int_{-\infty}^{\infty}\frac{d\omega}{2\pi}J(\omega)\sin(\omega t) \equiv a(t) - ib(t) \tag{S6}$$

is given in terms of the standard bath spectral density $J(\omega)$ and has the real part $a(t)$ and imaginary part $b(t)$. After inserting Eqs. (S4) and (S5) into Eq. (S3), we can express the last three terms of Eq. (S3) in terms of $a(t)$ and $b(t)$ in form

$$\begin{aligned} \mathcal{L}_{\rm s}^{\rm eff} &= \mathcal{L}_{\rm s} + \lambda^2\mu\mathcal{L}_{\rm ren,s} + \lambda^2\chi\mu\mathcal{L}^-, \\ K(t,t') &= \lambda^2\mathcal{L}^-\left(a(t-t')\mathcal{T}e^{\int_{t'}^t \mathcal{L}_{\rm s}}\mathcal{L}^- + b(t-t')\mathcal{T}e^{\int_{t'}^t \mathcal{L}_{\rm s}}\mathcal{L}^+\right), \\ \Gamma(t) &= \lambda^2\mathcal{L}^-\int_{-\infty}^0 dt'\left[a(t-t')\mathcal{T}e^{\int_{t'}^t \mathcal{L}_{\rm s}}\mathcal{L}^-\rho_{\rm s}^{\rm eq} + b(t-t')\mathcal{T}e^{\int_{t'}^t \mathcal{L}_{\rm s}}\mathcal{L}^+\rho_{\rm s}^{\rm eq}\right]\,, \end{aligned} \tag{S7}$$

with $\mathcal{L}^- = -i[H_{\rm sb}, \cdot]$ and $\mathcal{L}^+ = [H_{\rm sb}, \cdot]_+ - 2\chi$. The potential renormalization is given in terms of the spectral density as $\mu = \int_{-\infty}^{\infty}\frac{d\omega}{2\pi}J(\omega)/\omega$.

In order to obtain a closed analytic form of the bath correlation function, any given spectral density (in our particular case, we use the standard Ohmic form) can be approximated by a sum of Lorentzian-like spectral terms [8, 9] according to the decomposition

$$J(\omega) = \frac{\pi}{2}\sum_{k=1}^n \frac{p_k\omega}{[(\omega+\Omega_k)^2 + \Gamma_k^2][(\omega-\Omega_k)^2 + \Gamma_k^2]}\,. \tag{S8}$$

The spectral amplitudes p_k, the frequencies Ω_k and the widths Γ_k follow from the expansion of the original function in terms of the Lorentzian shapes. Inserting the expanded form of $J(\omega)$ into Eq. (S6) results in

$$\begin{aligned} a(t) &= \sum_{k=1}^n \frac{p_k}{8\Omega_k\Gamma_k}\coth\left[\frac{\beta}{2}\left(\Omega_k + i\Gamma_k\right)e^{i\Omega_k t - \Gamma_k t}\right] + \sum_{k=1}^n \frac{p_k}{8\Omega_k\Gamma_k}\coth\left[\frac{\beta}{2}\left(\Omega_k - i\Gamma_k\right)e^{-i\Omega_k t - \Gamma_k t}\right] + \frac{2i}{\beta}\sum_{k=1}^{n'} J(i\nu_k)e^{-\nu_k t}, \\ b(t) &= \sum_{k=1}^n \frac{ip_k}{8\Omega_k\Gamma_k}\left(e^{i\Omega_k t - \Gamma_k t} - e^{-i\Omega_k t - \Gamma_k t}\right)\,, \end{aligned} \tag{S9}$$

with the Matsubara frequencies given by $\nu_k = 2\pi k/\beta$.

Next, we rewrite the correlation functions as $a(t) = \sum_{k=1}^{n_r} \alpha_k^r e^{\gamma_k^r t}$ and $b(t) = \sum_{k=1}^{n_i} \alpha_k^i e^{\gamma_k^i t}$ with $n_i = 2n$, $n_r = 2n+n'$, where n' is the number of Matsubara frequencies used. Then, we define new auxiliary 'density matrices' which incorporate both memory effects and initial correlations according to

$$\begin{aligned}\rho_k^r(t) &= \lambda\left(\mathcal{T}e^{\int_0^t dt'\mathcal{L}_s} e^{\gamma_k^r t}\int_0^\infty dt' e^{\mathcal{L}_s t'} e^{\gamma_k^r t'}\mathcal{L}^- \rho_s^{eq} + \int_0^t dt' e^{\gamma_k^r (t-t')}\mathcal{T}e^{\int_{t'}^t L_s}\mathcal{L}^-\rho_s(t')\right),\\ \rho_k^i(t) &= \lambda\left(\mathcal{T}e^{\int_0^t dt'\mathcal{L}_s} e^{\gamma_k^i t}\int_0^\infty dt' e^{\mathcal{L}_s t'} e^{\gamma_k^i t'}\mathcal{L}^+ \rho_s^{eq} + \int_0^t e^{\gamma_k^i (t-t')}\mathcal{T}e^{\int_{t'}^t dt' L_s}\mathcal{L}^+\rho_s(t')\right).\end{aligned} \tag{S10}$$

The time-retarded Eq. (S3) (first term) can be then deconvoluted into a set of coupled first-order equations as

$$\begin{aligned}\dot{\rho}_s(t) &= \mathcal{L}_s^{eff}(t)\rho_s(t) + \lambda\left[\sum_{k=1}^{n_r}\alpha_k^r\mathcal{L}^-\rho_k^r(t) + \sum_{k=1}^{n_i}\alpha_k^i\mathcal{L}^-\rho_k^i(t)\right],\\ \dot{\rho}_k^r(t) &= (\mathcal{L}_s(t) + \gamma_k^r)\rho_k^r(t) + \lambda\mathcal{L}^-\rho_s(t), \quad k = 1,\ldots,n_r,\\ \dot{\rho}_k^i(t) &= (\mathcal{L}_s(t) + \gamma_k^i)\rho_k^i(t) + \lambda\mathcal{L}^+\rho_s(t), \quad k = 1,\ldots,n_i.\end{aligned} \tag{S11}$$

This set of coupled time non-local quantum master equations was used for the calculations of the quantum dynamics and the resulting spectra.

B. Linear absorption spectrum

We have used the first-order transition dipole moment correlation function to calculate the absorption and circular dichroism spectra defined by

$$I(\omega) \propto \omega \int_{-\infty}^{+\infty} dt e^{i\omega t}\langle \boldsymbol{\mu}(t)\boldsymbol{\mu}(0)\rangle_g. \tag{S12}$$

Here, $\boldsymbol{\mu}$ is the transition dipole moment and the subscript g refers to performing the trace over a thermally equilibrated bath distribution and $\boldsymbol{R}_{m,n}$ is the distance vector between the monomers m and n. The correlation functions can be calculated as $\langle \boldsymbol{\mu}(t)\boldsymbol{\mu}(0)\rangle_g = \mathrm{tr_S}\{\boldsymbol{\mu}\mathrm{tr_B}[e^{-iHt}\boldsymbol{\mu}\rho_g e^{iHt}]\}$.

C. Calculation of 2D electronic spectrum

For the calculation of two-dimensional electronic spectra, we have applied the equation of motion-phase matching approach (EOM-PMA) established in Ref. [10]. In the EOM-PMA, the induced polarization in the direction of the photon-echo signal is calculated by the simultaneous propagation of three auxiliary density matrices ($\rho_1(t)$, $\rho_2(t)$, and $\rho_3(t)$), each of which obeys a modified effective equation of motion according to

$$\begin{aligned}\dot{\rho}_1(t) &= -i[H_s - V_1(t,t_1) - V_2^\dagger(t,t_2) - V_3^\dagger(t,t_3), \rho_1(t)] - \Re(t)\rho_1(t),\\ \dot{\rho}_2(t) &= -i[H_s - V_1(t,t_1) - V_2^\dagger(t,t_2), \rho_2(t)] - \Re(t)\rho_2(t),\\ \dot{\rho}_3(t) &= -i[H_s - V_1(t,t_1) - V_3^\dagger(t,t_3), \rho_3(t)] - \Re(t)\rho_3(t),\end{aligned} \tag{S13}$$

where $V_\alpha(t,t_\alpha) = XAe^{-(t-t_\alpha)^2/2\Gamma^2}e^{i\omega t}$, X is the transition dipole operator, Γ is the pulse duration, and $\Re$ is a relaxation superoperator. All three above master equations were calculated by adopting the TNL method Eq. (S11) to the auxiliary density operators with the corresponding different time-dependent Hamiltonians. Then, the third-order induced polarization is obtained as

$$P_{PE}(t_1,t_2,t_3,t) = e^{i\mathbf{k_s}\cdot\mathbf{r}}\langle X(\rho_1(t) - \rho_2(t) - \rho_3(t))\rangle + c.c., \tag{S14}$$

where the brackets $\langle\ldots\rangle$ indicate the evaluation of the trace.

The total 2D Fourier-transformed spectrum is then given by the double Fourier transform of the photon-echo polarization signal with respect to the delay time $\tau = t_2 - t_1$ and t according to

$$S_{PE}(\omega_\tau, T, \omega_t) \sim \int_{-\infty}^{+\infty} d\tau \int_{-\infty}^{+\infty} dt e^{-i\omega_\tau\tau} e^{i\omega_t t} P_{PE}(\tau, T, t). \tag{S15}$$

Here, ω_τ is the "coherence" frequency, ω_t is the detection frequency, and T is "waiting" time given by the difference between t_3 and t_2.

For the concrete simulations of the 2D spectra, a multi-processing interface (MPI) has been used to minimize the simulation time. The coherence time window $[-300\text{ fs}, 300\text{ fs}]$ was separated into time slices of length $d\tau$=2 fs and each specified time was sent to one CPU core for the calculation. Furthermore, 500 realizations have been used to account for the static disorder and the average of the interaction between the laser and the molecular transition dipole moment.

V. MODEL HAMILTONIAN

In this section, we describe the model Hamiltonian used for the calculation of the 2D spectra of the ion-pair. The total Hamiltonian is separated into the system and environmental part, i.e.,

$$H = H_{\text{mol}} + H_{\text{env}}, \tag{S16}$$

where H_{mol} is the molecular Hamiltonian and H_{env} is the part to describe the dissipation from the environment. In the molecular part, the electronic state of the F_4TCNQ^- anion interacts the PBTTT cation via Coulomb coupling. Additionally, to fit the feature of the vibrational progression observed in the absorption spectrum, one effective mode has been assumed which couples linearly to the electronic state of F_4TCNQ^-. Thus, the molecular Hamiltonian can be written as

$$H_{\text{mol}} = |g\rangle\,\Omega(\alpha^\dagger\alpha + \frac{1}{2})\,\langle g| + |e_F\rangle\left[E_F + \alpha^\dagger\alpha + \frac{1}{2} + \lambda(\alpha^\dagger + \alpha)\right]\langle e_F| + |e_P\rangle\,E_P\,\langle e_P| + (|e_F\rangle\,V\,\langle e_P| + H.c.)\,. \tag{S17}$$

Here, $|g\rangle$, $|e_F\rangle$ and $|e_P\rangle$ denote the electronic ground state and the excited states of F_4TCNQ^- and $PBTTT^+$, respectively. Ω is the vibrational frequency of the effective mode which vibronically couples to the electronic state $|e_F\rangle$ with the coupling strength λ. E_F and E_P are the site energies of the cation PBTTT and the anion F_4TCNQ, which are excitonically coupled with the strength V.

For simplicity, we assume that the dissipative fluctuations are generated by an infinity number of harmonic oscillators with the Hamiltonian

$$H_{\text{env}} = \sum_{i=F/P}\sum_j\left[\frac{p_j^{(i)2}}{2m_j^{(i)}} + \frac{m_j^{(i)}\omega_j^{(i)2}}{2}\left(x_j^{(i)} + \frac{c_j^{(i)}\,|e_i\rangle\langle e_i|}{m_j^{(i)}\omega_j^{(i)2}}\right)^2\right]. \tag{S18}$$

Here, the momenta of the bath oscillators are denoted by $p_j^{(i)}$, while their coordinates, masses and vibrational frequencies are denoted by $x_j^{(i)}$, $m_j^{(i)}$ and $\omega_j^{(i)}$, respectively. We assume that the two excited states each couple to their own bath, but the two baths are assumed to have equal characteristics. The coupling strengths between system and baths are denoted as $c_j^{(i)}$. The baths are characterized by the Ohmic spectral densities $J_{F/P}(\omega) = \eta_{F/P}\omega\exp(-\omega/\omega_{c,F/P})$ with the coupling strengths $\eta_{F/P}$ and the cutoff frequencies $\omega_{c,F/P}$.

On the basis of this model Hamiltonian, we calculate the linear absorption and 2D electronic spectra. To fit the experimental results, we optimize the parameters as follows. First, the site energies are calculated based on a quantum chemistry calculation and then further optimized based on the fitting to the measured data, $e_F = 11450$ cm^{-1} and $e_P = 12300$ cm^{-1}. The frequency of the effective mode is obtained from the absorption spectrum of F_4TCNQ [11] as $\Omega = 1500$ cm^{-1}. The dimensionless displacement Δ is refined and optimized by fitting of the vibrational progression. This yields $\Delta = 0.528$, and by this, the coupling strength $\lambda = \frac{\Omega\Delta}{\sqrt{2}} = 560$ cm^{-1}. In order to obtain the stable eigenbasis sufficient for numerical convergence, 8 vibrational levels are included to construct the Hamiltonian matrix. Based on the fitting, we refine the strength of the electronic coupling to the value $V = 250$ cm^{-1} according to the parallel transition dipole moments of the ion-pair, which was proposed in the Ref. [3]. To fit the line broadening in the absorption and 2D spectra, the parameters of the spectral density are optimized. We find $\eta_F = 1.0$, $\omega_{c,F} = 400$ cm^{-1} for the anion F_4TCNQ and $\eta_P = 1.0$, $\omega_{c,P} = 800$ cm^{-1} for the cation PBTTT. In addition, inhomogeneous broadening is included by site energies which are Gaussian distributed. We have $\Delta E_F = 70$ cm^{-1} and $\Delta E_P = 350$ cm^{-1}. Moreover, in order to account for the ultrafast deactivation dynamics between the excited state of F_4TCNQ and the electronic ground state, the Lindblad equation is applied to mimic the decay dynamics of the electronic wave packet via the conical intersection. The Lindblad damping constant $\gamma = 50$ fs is assumed for the vibrational damping of all the vibronic states in F_4TCNQ to the ground state to fit the kinetics revealed in the measurements.

VI. LIFETIME OF THE ELECTRONIC COHERENCE BETWEEN CATION AND ANION

In this section, we show the kinetic trace of the peak E (as labeled in the main text) and the corresponding fitting trace. Both are shown in Fig. S3. It clearly shows strong and rapid dephasing and the electronic coherence completely disappears within the initial waiting time. We can determine the frequency resolved information by performing the Fourier transform of the residuals after subtracting the global exponential kinetics from the raw data. We find a broadband peak located at $\sim$750 cm^{-1} which clearly manifests the short lifetime of the electronic coherence, which caused by the strong static disorder of the PBTTT cation and the deactivation of the wave packet via the conical intersection of the excited state and the ground state of the F_4TCNQ anion.

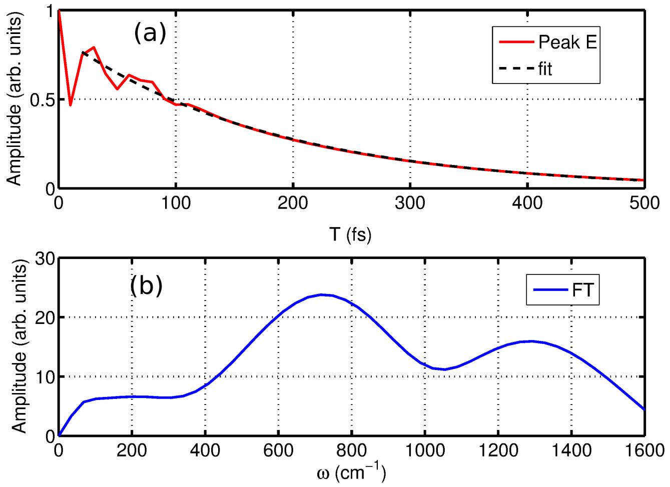

FIG. S3. (a) Kinetic trace (red) extracted for the peak E at (ω_τ, ω_t) = (12250, 13000) cm^{-1}, which is fitted by the black dotted line. (b) Oscillatory information superimposed on the kinetics obtained by performing the Fourier transformation of the residual after subtracting the exponential decay. One broad peak is observed at $\sim$750 cm^{-1}, which perfectly matches the electronic energy gap between peak B and C.

[1] M. J. Frisch, G. W. Trucks, H. B. Schlegel, G. E. Scuseria, M. A. Robb, J. R. Cheeseman, G. Scalmani, V. Barone, B. Mennucci, G. A. Petersson, H. Nakatsuji, M. Caricato, X. Li, H. P. Hratchian, A. F. Izmaylov, J. Bloino, G. Zheng, J. L. Sonnenberg, M. Hada, M. Ehara, K. Toyota, R. Fukuda, J. Hasegawa, M. Ishida, T. Nakajima, Y. Honda, O. Kitao, H. Nakai, T. Vreven, J. A. Montgomery, Jr., J. E. Peralta, F. Ogliaro, M. Bearpark, J. J. Heyd, E. Brothers, K. N. Kudin, V. N. Staroverov, R. Kobayashi, J. Normand, K. Raghavachari, A. Rendell, J. C. Burant, S. S. Iyengar, J. Tomasi, M. Cossi, N. Rega, J. M. Millam, M. Klene, J. E. Knox, J. B. Cross, V. Bakken, C. Adamo, J. Jaramillo, R. Gomperts, R. E. Stratmann, O. Yazyev, A. J. Austin, R. Cammi, C. Pomelli, J. W. Ochterski, R. L. Martin, K. Morokuma, V. G. Zakrzewski, G. A. Voth, P. Salvador, J. J. Dannenberg, S. Dapprich, A. D. Daniels, . Farkas, J. B. Foresman, J. V. Ortiz, J. Cioslowski, and D. J. Fox, Gaussian, Inc., Wallingford CT, 2009.

[2] Kistler, K. A. *et al.* A Benchmark of Excitonic Coupling Derived from Atomic Transition Charge. *J. Phys. Chem. B* **117**, 2032-2044 (2013).

[3] Cochran, J. E. *et al.* Molecular Interactions and ordering in electrically doped polymers: Blends of PBTTT and F_4TCNQ. *Macromolecules* **47**, 6836 (2014).

[4] Meier, C., & Tannor, D. J. Non-Markovian Evolution of the Density Operator in the Presence of Strong Laser Fields. *J. Chem. Phys.* **111**, 3365-3376 (1999).

[5] Kleinekathöfer, U. Non-Markovian Theories Based on a Decomposition of the Spectral Density. *J. Chem. Phys.* **121**, 2505-2514 (2004).

[6] Zwanzig, R. *Lectures in Theoretical Physics*, Boulder, Colorado (Interscience, New York, 1961), Vol. 3.

[7] Morillo, M. & Cukier, R. I. Controlling Low-temperature Tunneling Dynamics With External Fields. *Phys. Rev. B* **54**, 13962-13973 (1997).

[8] Kleinekathöfer, U. *et al.* Memory Effects in the Fluorescence Depolarization Dynamics Studied within the B850 Ring of Purple Bacteria. *J. Phys. Chem. B* **107**, 14094-14102 (2003).

[9] Ritschel, G. & Eisfeld, A. Analytic Representations of Bath Correlation Functions for Ohmic and Superohmic Spectral Densities Using Simple Poles. *J. Chem. Phys.* **141**, 141, 094101 (2014).

[10] Gelin, M. F. & Egorova, D. & Domcke, W. Efficient Method for the Calculation of Time- and Frequency-Resolved Four-wave Mixing Signals and Its Application to Photon-echo Spectroscopy. *J. Chem. Phys.* **123**, 123, 164112 (2005).

[11] Ma, L. *et al.* Ultrafast Spectroscopic Characterization of 7,7,8,8-tetracyanoquinodimethane (TCNQ) and its radical anion ($TCNQ^-$). *Chem. Phys. Lett.* **609**, 11-14 (2014).
